# Histopathological Insight of a Case of Adenoid Ameloblastoma: A Rare Odontogenic Tumor

**DOI:** 10.1155/2024/8366045

**Published:** 2024-04-29

**Authors:** Shashi Keshwar, Toniya Raut, Neetu Jain, Ashish Shrestha, Mehul Rajesh Jaisani

**Affiliations:** ^1^Department of Oral Pathology, College of Dental Surgery, B.P. Koirala Institute of Health Sciences, Dharan, Nepal; ^2^Department of Oral Pathology, College of Dental Surgery, Universal College of Medical Sciences, Siddharthanagar, Nepal; ^3^Department of Oral and Maxillofacial Surgery, College of Dental Surgery, B.P. Koirala Institute of Health Sciences, Dharan, Nepal

## Abstract

Adenoid ameloblastoma with dentinoid had been perceived as a rare odontogenic tumor with bridging histopathological features between ameloblastoma and adenomatoid odontogenic tumor. Due to the mixture of histopathological features of two separate and well-recognized entities, adenoid ameloblastoma was also regarded as a hybrid lesion. The diversity in the histopathological presentation among the cases has disaccorded the nature, behaviour, and prognosis of this pathology. Despite the literature acknowledging the histopathological diversity, categorizing all these variations into one and addressing them as a single entity was lagging till the 5^th^ edition of the odontogenic tumor classification by the WHO was forwarded. With the establishment of the new terminology of adenoid ameloblastoma and the enlistment of its diagnostic criteria, the scientific literature has advocated updating, contributing, and redefining the various aspects of this pathology. Here, we present a case of a 34-year-old male who presented with a chief complaint of swelling in the lower front region of his jaw in the past one month. The swelling was associated with pain that was sudden in onset with a progressive increase in size. The swelling was also associated with discharge that resembled pus. A panoramic radiograph showed a mixed radiopaque and radiolucent area, extending from the distal aspect of 32 up to the distal aspect of 43. The entire cystic lining along with the growth was excised and sent for histopathological examination. Correlating clinically, the histopathological features are suggestive of adenoid ameloblastoma. Scientific literature has stood as a boon to evidence-based practice. The diagnosis for the present case report is truly an outcome of the literature-based update which helped the diagnosis of the case as a separate entity rather than as a hybrid pathology. The goal was to enhance the understanding of the lesions in terms of their clinical characteristics and diverse histopathological morphology.

## 1. Introduction

Adenoid ameloblastoma is a benign epithelial odontogenic tumor, composed of cribriform architecture and duct-like structures, and frequently includes dentinoid [1]. The lesion was first witnessed by Slabbet et al. in 1992 as dentino ameloblastoma [2]. After which, various authors encountered similar histopathological presentation with some other desirable features, opting for various terminologies such as adenomatoid odontogenic tumor originating within unicystic ameloblastoma, atypical ameloblastoma with dentinoid, hybrid ameloblastoma, ameloblastoma with features of dentinoid, and atypical adenoid ameloblastoma [3–8]. The term adenoid ameloblastoma with dentinoid was first proposed by the Armed Forces Institute of Pathology in 1994 by Brannon [9]. Now, it has been universally accepted and adopted by the 5^th^ edition of the WHO classification for odontogenic tumors [1]. The word “ameloblastoma” from adenoid ameloblastoma is justified histopathologically by the presence of ameloblast-like cells showing intense, focal expression of calretinin, a specific marker for neoplastic ameloblastic epithelium [10]. However, these ameloblast-like cells fail to express BRAF p.V600E, the most common activating mutation seen in mandibular ameloblastoma [11, 12]. The pathology was categorized as a separate entity of benign epithelial odontogenic tumor rather than a subvariant of ameloblastoma [13].

As per the literature review, adenoid ameloblastoma often presents as a painless swelling affecting a wide age range between the 2^nd^ and 5^th^ decades of life, predominately in the mandibular anterior region, with a slight predominance in females. Ill-defined radiolucency with cortical perforation is the common radiographic feature associated with the lesion. Histopathological pictures encompass the combined features of ameloblastoma, predominantly plexiform variant, ductal component, and whorl or rosette pattern corresponding to adenomatoid odontogenic tumor (AOT), along with dentinoid. In addition, clear cells and ghost cells have also been associated with it [1, 14]. The major concern about adenoid ameloblastoma is its biological behaviour. The existing literature says that adenoid ameloblastoma is a locally aggressive odontogenic tumor with a high recurrence rate ranging from 45.5 to 70% [8, 10, 14, 15]. Loyola et al. reported one of his cases showing a maximum of nine recurrences over a period of 19 months. An increase in the number of reoccurrences is seen to be associated with the cases that were underdiagnosed as AOT with a conservative approach to the treatments and also in maxillary pathology due to inadequate margins [4, 8]. The high recurrence rate has also been justified by the presence of clear cell components and some degree of cellular atypia. A higher Ki-67 index also explains the aggressive behaviour showing a high recurrence rate [8].

## 2. Case Report

A 34-year-old male presented with a chief complaint of swelling in the lower front region of the jaw in the past one month. The swelling was associated with pain that was sudden in onset with a progressive increase in size. The swelling was also associated with discharge that resembled pus. The patient had a similar history for the same site three months back which subsided on its own after discharge.

He also had a history of trauma in the front region of the face 18 years back which was followed by a dental check-up and medication (antibiotic) after which it remained uneventful. The patient did not have any other significant medical history.

The patient had undergone root canal treatment in relation to 31, 41, and 42, with crown placement in relation to 11.

On examination, there was a single, localized, oval swelling of approximately 3 × 2.5 *cm*^2^ in maximum dimension. The swelling was firm in consistency and tender on palpation. It extended from the 33 to 43 region on the labial aspect of the anterior mandible, along with a lingual bulge in relation to the 41 region. There was tenderness on vertical percussion in relation to 31, 32, 41, and 42 and restored teeth in relation to 31, 32, and 41 without any discharge or bleeding on palpation. On chair-side evaluation with an electric pulp tester, 32, 33, 34, 43, and 44 showed responses at a level of “3.” On aspiration with a wide-bore needle, straw-colored fluid with a blood-tinted appearance was seen.

**Figure 1:**
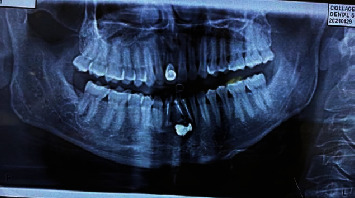
Panoramic radiograph showing mixed radiopaque and radiolucent area in relation to 32-43.

A panoramic radiograph showed a mixed radiopaque and radiolucent area, extending from the distal aspect of 32 up to the distal aspect of 43. It also exhibits dense irregular radiopaque mass (probably extruded root canal filing agent) and root canal filling within the root canal and periapical regions of 31, 41, and 42. (Figure 1).

With a clinical diagnosis of a radicular cyst and a differential diagnosis of the calcifying odontogenic cyst, a biopsy was conducted, which showed the cystic lining intraoperatively. The entire cystic lining along with the growth was excised and sent for histopathological examination.

**Figure 2:**
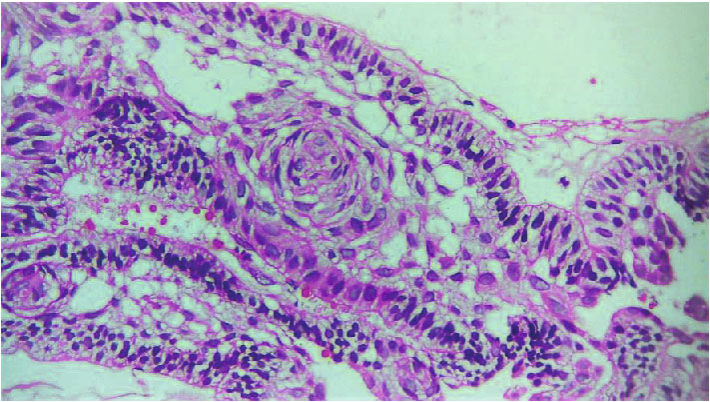
Section of tissue showing follicles with ameloblast-like cells (40x).

**Figure 3:**
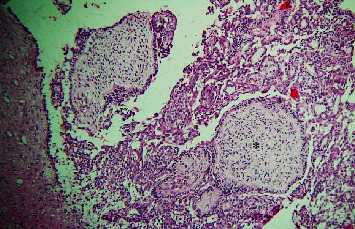
Section of tissue showing dental follicle-like structure showing primitive mesenchyme (marked as ∗) like component (10x).

**Figure 4:**
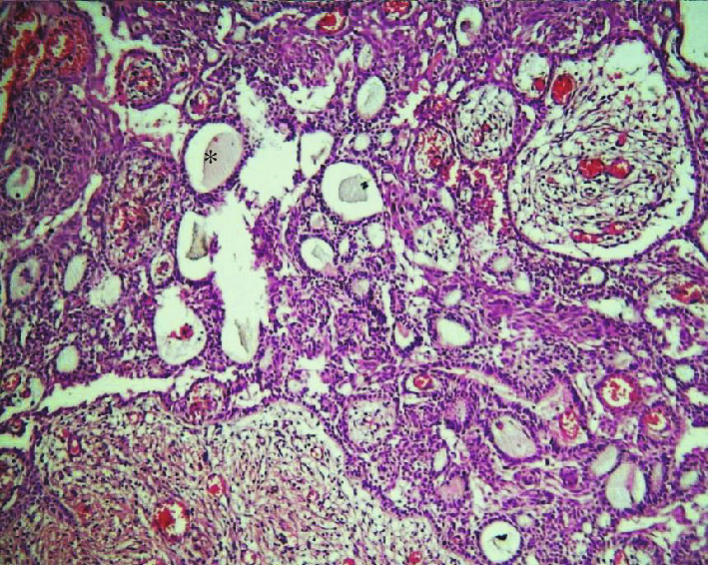
Section showing ductal component and central area of eosinophilic content (marked as ∗) (10x).

**Figure 5:**
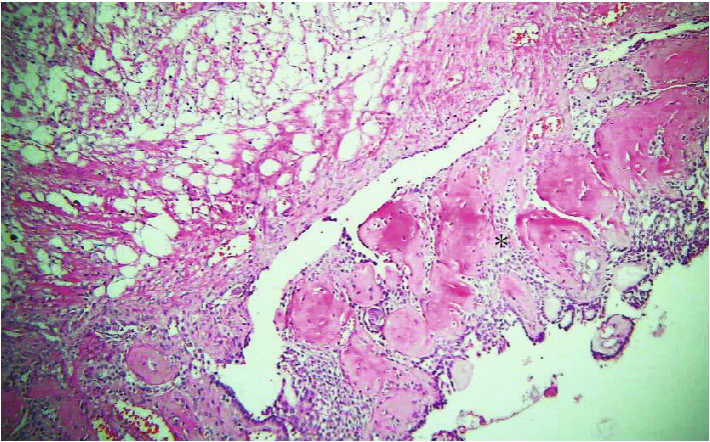
Section shows dentinoid component (marked as ∗) (4x).

**Figure 6:**
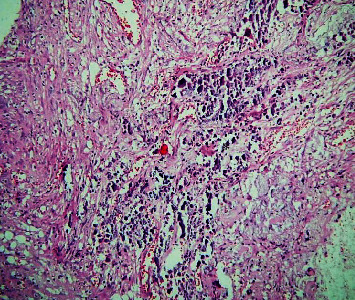
Dystrophic calcified deposits (marked as ∗) (4x).

The histopathological section of the lesion shows areas of interlacing strands and cords lined peripherally by tall columnar cells with hyperchromatic nuclei arranged in a palisading pattern and subnuclear vacuolization that surrounds loosely cellular stellate reticulum-like cells (Figure 2). Numerous areas of dental follicle-like structures with primitive mesenchyme-like components were seen (Figure 3). Areas of polygonal cells in whorl-like patterns along with duct-like structures lined by a single layer of cuboidal to polygonal cells with a central area of eosinophilic content are also seen (Figure 4). Multiple areas of calcified dentin-like structures along with dystrophic calcification were also evident (Figures 5 and 6). The surrounding connective tissue was highly vascular, with numerous dilated endothelial cells lining blood vessels and a few inflammatory cells infiltrating predominantly lymphocytes and plasma cells. A definitive diagnosis of adenoid ameloblastoma was made based on the clinical, radiographical, and histopathological findings. The postoperative period was uneventful. The patient was advised for routine follow-up, but the patient failed to follow up.

## 3. Discussion

Ameloblastoma is a most commonly occurring benign, slow-growing, locally aggressive odontogenic tumor with a high recurrence rate. Histologically, it is characterized by the presence of peripherally arranged ameloblast-like cells and centrally placed stellate reticulum-like cells [16]. WHO classified it as conventional, unicystic, peripheral, and metastasizing type. Conventional is further categorized histologically as plexiform, follicular, acanthomatous, granular, basal, and desmoplastic types [17]. An adenomatoid odontogenic tumor (AOT) is a relatively rare, distinct benign odontogenic tumor with indolent behaviour and a rare recurrence. Histologically, it is characterized by the presence of spindle-shaped cells or polygonal cells arranged in sheets, whorls, or rosette patterns along with duct-like structures and inductive changes [18, 19]. Adenoid ameloblastoma is a distinct pathological entity showing features that correspond to both ameloblastoma and AOT in all the spectra of clinical, radiographical, histopathological, and biological behaviour. The essential diagnostic criteria have been summarised as lesions occurring in the 4^th^ decade of life with slight male predilection and no site predilection. The histopathological criteria are ameloblast-like components, duct-like structures, whorls/morules, and cribriform architectures which correspond to the findings in our case [1].

In the present case report, we have compiled the major findings associated with adenoid ameloblastoma based on the accessible case report with adequate information available in the literature (Table 1). This includes a total of 40 reported cases of adenoid ameloblastoma that correspond to the histopathological features of adenoid ameloblastoma. According to this compilation, adenoid ameloblastoma has a very wide range of age distribution ranging from the 1^st^ decade to the 8^th^ decade of life. The maxilla and mandible are equally affected, with a significant incidence in the posterior aspect of the jaw. The present case reports a lesion in the anterior aspect of the jaw.

The histopathological presentation of adenoid ameloblastoma shows wide variations. The ameloblastic component varies as plexiform, follicular, or unicystic type; a plexiform pattern was seen in the present case. Polygonal cells in a whorl pattern along with a duct-like structure indicate the AOT-like features. Dentinoid-like materials are also seen to resemble the present case [10]. The polygonal tumor islands may show ghost cells and clear cells. A systematic review conducted by De Farias Morais et al., with 30 cases of adenoid ameloblastoma, showed the presence of a plexiform variant in 50% of the cases, a cribriform pattern in 90% of the cases, a whorl and duct-like pattern in 100% of the cases, and a dentinoid in 70% of the total cases [37]. Several special stains, like alcian blue, periodic acid Schiff (PAS), and musicarmine, have been used to highlight duct-like structures in the section [3, 33]. Van Gieson's stain highlights ghost cells [26].

Incisional biopsy, as recommended in many of the large-size lesions for the diagnosis, is often predominated either by an ameloblast-like cell or an AOT-like whorl or ductal pattern. This often leads to the misdiagnosis of adenoid ameloblastoma as ameloblastoma or AOT [10]. Hence, a critical analysis to determine the traits of each component in the entire specimen would help to prevent its misdiagnosis. Adenoid ameloblastoma shows aggressive behaviour with a very high recurrence rate, yet the adapted treatment approach varies from conservative to aggressive surgical resection. Recurrence has been reported despite aggressive treatment [8]. Recurrence has also been noticed to occur over a period of long gaps [4, 8], emphasizing a mandatory long-term follow-up and close evaluation. Our study has the limitation of loss of follow-up.

Molecular studies have confirmed adenoid ameloblastoma to be a separate entity by expressing nuclear accumulation of *β*-catenin and an altered WNT pathway [13, 38]. In addition, it has also denied itself as a hybrid tumor corresponding to the features of ameloblastoma and AOT, as shown by the absence of the BRAF V600E and *KRAS* p.G12V mutations [39]. The biological behaviour of adenoid ameloblastoma has been evaluated by Ki-67, a proliferative marker. The result varies from strong positivity [8] to weak positivity [33]. Despite the variation, the strong positivity in the case series by Loyola et al. can be correlated to the aggressive nature, as shown by multiple recurrences. Similarly, weak positivity in the case of De Arruda et al. correlates to nonaggressive behaviour, as shown by no evidence of recurrence.

Uncommon pathologies are often missed because of their limited incidence; adenoid ameloblastoma is one of those pathologies. In addition, its histopathological features that conjoin features of multiple pathologies have made it more deceiving.

The distinct entities constituting this lesion are frequently misdiagnosed as ameloblastoma, AOT, or other similar odontogenic tumors presenting the equivalent features, and the quantity of one component in an incisional biopsy overshadows the other component. In many cases, this leads to a misdiagnosis of the lesion, as AOT and conservative treatment result in recurrence. To rule out such mixed tumor presentation, it is critical to determine the traits of each component in the histopathological specimens of the odontogenic tumor.

Since we are now more committed to evidence-based practice, we must keep ourselves updated with recently published literature. This calls for the sharing of information and experience in the scientific community, which is what we accomplished with this case study. This would not only introduce the uncommon pathology to the world, but it would also help the oral healthcare professional decide on the best course of action for the patient's care.

## 4. Conclusion

Adenoid ameloblastoma is a distinct new pathological entity. The histopathological presentation establishes a strong background for the diagnosis; however, it requires an updated knowledge of the scientific literature. In the present era, evidence-based practice is a boon to diagnose a lesion which is uncommon in presentation yet lagging in identification. Literature writing in scientific journals is the basis for evidence-based practice, and we believe that our case will also contribute to disseminating knowledge on adenoid ameloblastoma. Molecular analysis and loss of follow-up were the limitations of our case study. However, we would recommend maintaining a long-term follow-up. With the availability of setup, the use of a proliferative marker like Ki-67 would contribute to correlating the biological behaviour of individual cases that would direct the treatment approach, thereby decreasing the chance of recurrence.

## Figures and Tables

**Table 1:** Tabulation of case report for adenoid ameloblastoma.

Year	Author	Sex/age	Site/side	Treatment/no. of recurrence	No. of follow-up (months)
1959 [20]	Waldron	F/79	Mn, Ant.	SR; 3 rec	—

1978 [21]	de Andrade Sobrinho et al.	F/41	Mn, Post.	SR; 1 rec	—

1985 [22]	Takata et al.	M/71	Max, Post.	SR	16

1992 [23]	Tajima et al.	M/35	Mn, Ant	SR	60

1992 [2]	Slabbert et al.	M/24	Mn, Lf	WE	—

2001 [3]	Matsumoto et al.	M/19	Mn, Rt	Marsupialization-enucleation; 1 rec after 2 yrs-WE	30

2004 [4]	Evans et al.	M/39	Mn, bilateral	WE; WE-curettage; enucleation-curettage; 3 rec in 16 yrs; SR	18

2006 [5]	Zhang et al.	F/64	Mn, bilateral	SR	36

2007 [7]	Jivan et al.	M/40	Mn, bilateral	—	—

2008 [24]	Ghasemi-Moridani and Yazdi	F/19	Max, Rt	Excision	—

2009 [25]	Ide et al.	M/44	Max, bilateral	Enucleation-extraction; 3 rec in 11 yrs; partial maxillectomy	96

2011 [26]	Sonone et al.	F/35	Mn, Rt	SR	6

2012 [27]	Saxena et al.	M/45	Max, Lf	Enucleation; SR; 3 rec; subtotal maxillectomy	—

2013 [28]	Kumar et al.	M/55	Mn, Rt	SR	36

2014 [6]	Yamazaki et al.	F/31	Mn, Rt	SR	36

2015 [8]	Loyola et al.	M/55	Mn, Post.	SR; 1 rec	108
		F/34	Max, Post	SR; 9 rec	19
		F/33	Max, Post	SR-Radio; 5 rec	76
		M/51	Max, Ant.	SR; 5 rec	282
		M/47	Max, Post	SR-radio; 2 rec	52

2016 [15]	Khalele et al.	M/40	Mn, Rt	Hemimandibulectomy	14

2016 [29]	Salehinejad et al.	F/34	Max, Rt	9 rec; SR	19

2017 [30]	Rai et al.	M/55	Mn, Rt	Enucleation	—

2017 [31]	Sathyanarayan et al.	M/51	Max, bilateral	5 rec; SR	76

2018 [32]	Adorno-Farias et al.	F/34	Mn	SR	—
		F/-	—	SR	—
		M/15	Mn	SR	—
		M/82	Mn, post.	SR	—
		M/46	—	SR	—
		F/15	Mn, Ant.	SR	—
		M/37	Mn, Post	SR	—
		46/F	Mn	SR	—

2020 [33]	De Arruda et al.	F/54	Max, Lf	SR	36

2022 [14]	Jayasooriya et al.	F/38	Mn, Lf	Excision; rec after 4 yrs; Hemimandibulectomy	—
		F/40	Mn	-/3 rec-6 yrs	—
		F/42	Max, Lt	Excision	6

2023 [34]	Silver et al.	M/13	Max, Lf	Partial maxillectomy	—

2023 [35]	Jabbar et al.	F/22	Mn, Lf	Incomplete excision	—
		M/49	Max, Lf	2 rec	—

2023 [36]	Chettiankandy et al.	F/35	Mn, Lf	Conservative excision	24

F: female; M: male; Max: maxilla; Mn: mandible; Rt: right; Lf: left; SR: surgical resection; WE: wide excision; Rec: reoccurrence; Yrs: years; —: data not available.

## Data Availability

Data will be available on demand.
